# PEX-168 improves insulin resistance, inflammatory response and adipokines in simple obese mice: a mechanistic exploration

**DOI:** 10.1186/s12902-021-00908-1

**Published:** 2021-12-20

**Authors:** Zeyuan Guo, Yuting Wu, Lihua Zhu, Yong Wang, Daorong Wang, Xiaofang Sun

**Affiliations:** 1 College of Nursing, Yangzhou University, Yangzhou, China; 2 Northern Jiangsu People’s Hospital, Yangzhou, China; 3 General Surgery Institute of Yangzhou, Yangzhou University, Yangzhou, China; 4 Clinical Medical College of Yangzhou University, Yangzhou, China

**Keywords:** GLP-1, weight loss, chemerin, omentin, Homa-IR, CRP

## Abstract

**Background:**

Polyethylene glycol loxenatide (PEX-168) is a new antidiabetic drug; as such, there are not yet any reports on its weight loss effect. Therefore, this trial was designed to investigate the effect of PEX-168 on simple obese mice.

**Methods:**

Thirty healthy male C57BL/6 mice were randomly selected and divided into a control group (NC) and an obesity model group. The high-fat diet-induced simple obesity mice were divided into a model control group (HF) and three intervention groups. The intervention groups were injected with different doses of PEX-168 intraperitoneally once a week for 12 weeks (low (LD), medium (MD) and high (HD)). Fasting blood glucose (FBG), body weight and food intake were measured from 1 to 12 weeks after PEX-168 injection. The serum insulin (INS), C-reactive protein (CRP), chemerin and omentin levels were measured after 12 weeks.

**Results:**

Compared with the HF group, the low dose of PEX-168 reduced the body weight of the mice in a short period of time (8 weeks), and the mice in the MD and HD groups showed a significant decrease in body weight (*P* < 0.05). The low dose of PEX-168 could effectively improve the blood glucose and homeostasis model assessment of insulin resistance (Homa-IR) of the mice (FBG *P* < 0.05 INS, Homa-IR *P* < 0.001), but there was no significant difference between different doses (*P* > 0.05). CRP levels in the MD and HD groups were significantly improved (*P* < 0.05). The levels of serum chemerin and omentin in the intervention groups were also significantly improved (*P* < 0.01), but there was no significant difference between the different doses (*P* > 0.05).

**Conclusions:**

PEX-168 significantly reduced the body weight of simple obese mice and improved the insulin resistance. PEX-168 may regulate the expression of chemerin and omentin through its hypoglycaemic effect, and the weight-reducing effect of PEX-168 is unlikely to be the reason for the changes in both.

## Introduction

The incidence rate of obesity has doubled in recent decades, which has caused health challenges among the world population [1]. Obesity is not only a risk factor of cardiovascular disease and type 2 diabetes mellitus [2], but also increases the financial burden of medical care [3]. Lifestyle (e.g., diet and exercise) and behaviour changes are the cornerstones of obesity management [4], but they are difficult for patients to comply with and maintain, and effective noninvasive treatments are limited[5]. The risks of bariatric surgery are high, and the surgery is only suitable for a small group of patients [6]. Considering the huge financial burden caused by obesity, the cost of interventions that can sustain weight loss is also critical. Therefore, new treatment options are of great significance for overweight and obese patients without type 2 diabetes to better manage their weight, and prevent or improve insulin resistance and inflammation, thereby reducing the risk of diabetes and improving their quality of life [7].

GLP-1 is a peptide substance that has a series of physiological functions such as promoting insulin release, promoting the proliferation and differentiation of pancreatic β-cells, increasing satiety and reducing food intake, and has a low risk of hypoglycaemia [8]. Many studies have demonstrated that overweight or obese people with type 2 diabetes have a better tolerance to the GLP-1 receptor agonist liraglutide, which can reduce food intake without increasing energy consumption, to achieve a good weight loss goal [9, 10]. However, due to the action of dipeptidyl peptidase IV, the half-life of GLP-1 in vivo is short, and several repeated doses are required to achieve therapeutic effects. Compared with natural GLP-1, although liraglutide is not easily and rapidly degraded by dipeptidyl peptidase IV in vivo, its half-life is also only 13 h, and it still needs to be injected subcutaneously once a day, which affects patient compliance. PEX-168 is modified by amino acids and pegylation of the chemical structure of Exenatide, which is the first long-acting GLP-1 receptor agonist (GLP-1 RA) developed independently in China, and has entered the Chinese health insurance system. Its introduction for the treatment of obesity can significantly reduce medical costs. PEX-168 becomes a long-acting formulation through the modification of polyethylene glycol, and only needs to be administered once a week, thus increasing patient compliance.

However, research in Jessica and Tina pointed out that further research is still needed to determine the effect of GLP-1 receptor agonists in overweight or obese patients without type 2 diabetes [11, 12]. Studies have proven the efficacy and safety of PEX-168 in the treatment of patients with T2DM [13, 14], but the effect of PEX-168 on simple obesity and on the regulation of chemerin and omentin expression has not been reported. This study therefore selected six indicators to evaluate the effect of PEX-168 according to prior research and the drug characteristics. Serum omentin and chemerin are newly discovered novel adipokines. As an inflammatory chemokine and adipokine, chemerin is considered to be a common factor between obesity and T2DM [15]. Recent studies have shown that plasma levels and gene expression of omentin-1 are negatively associated with obesity and insulin resistance [16]. Both are closely related to obesity and disorders of glycolipid metabolism and can reflect the hyperlipidaemia and inflammatory state of patients. Obesity is a significant determinant of insulin resistance, and adipose tissue plays a key role in insulin resistance [17]; insulin resistance is a common pathophysiological condition of obesity and diabetes [18]. Diet-induced obesity is associated with chronic low-grade inflammation of the hypothalamus. Metabolic inflammation is also a significant feature of obesity. Obesity is associated with an increased risk of cardiovascular disease (CVD), and the risk factor is modifiable [19]. CRP is now one of the most powerful predictors of inflammatory and lipid markers of cardiovascular events [20]. Obesity, inflammation and insulin resistance are closely related; obese patients without dyslipidaemia, insulin resistance, inflammatory state, and impaired fasting blood sugar have a very low risk of developing diabetes [21].

Therefore, the aim of this study was to observe the effects of different doses of PEX-168 on body weight, intake, Homa-IR, CRP, and the adipokines chemerin and omentin in simple obese mice induced by a high-fat diet, to observe whether there was variability between different doses and to provide a basis for subsequent clinical studies.

## Materials and methods

### Reagents and materials

Long-acting glucagon-like peptide-1 analogue polyethylene glycol losenatide (PEX-168) was provided by Hausen Pharmaceutical Co., Ltd (Jiangsu, China), lot number: H20190024, specification: 0.5 ML (0.1 mg) per bottle, stored at 4~8℃, Elisa kit was purchased from Liko Biotechnology Co., Ltd (Nanjing, China), precision electronic balance was purchased from Shuangjie Electronics Co., Ltd (Jiangsu, China). Automatic biochemical analyzer (Hitachi, Japan). Glucometer (Roche, Germany).

The basic diet and high-fat diet were purchased from Yangzhou University College of Veterinary Medicine (Jiangsu, China) and Medison Biomedical Co., Ltd (Jiangsu, China) respectively. The basic diet was composed of 22.8% protein, 13.8% fat, and 63.4% carbohydrate, the high-fat diet was composed of 26.2% protein, 34.9% fat, and 26.3% carbohydrate. The caloric density was 3656 kcal/kg for the basic diet and 5240 kcal/kg for the high-fat diet.

### Animals

#### Animal model and test methods

Thirty six-week-old SPF-grade male C57BL/6 mice were purchased from the College of Agriculture, Yangzhou University, and housed in cages at room temperature between 18 and 24 °C. They were first fed normal chow for one week for acclimation and exposed to light for 12 H daily, with unlimited access to chow and water. This experiment was approved by the Animal Ethics Committee of Yangzhou University. Starting from the age of seven weeks, they were randomly divided into five groups, and one group (NC group, *n *= 6) was randomly selected separate from the five groups as a control group and continued to be fed with ordinary feed, and the rest of the groups were fed high-fat feed to establish an obesity model until the weight of the obesity model group exceeded 20% of the control group, and there was no difference in body weight between the obesity groups, meanings that the model is successful.

After successful modeling, the four obesity model groups were randomly divided into three intervention groups: low dose (0.03 mg/kg) group (LD group, *n *= 6), medium dose (0.1 mg/kg) group (MD group, *n *= 6), high dose (0.33 mg/kg) group (HD group, *n *= 6) and an obese control group (HF group, *n *= 6).

After grouping, the three intervention groups and the obese control group transitioned from a high-fat diet to a normal diet after one week. The intervention started at the end of the dietary transition period and lasted for 12 weeks. The intervention groups were injected intraperitoneally with different doses of PEX-168, according to their specific subgroup, once a week at 8 a.m. on Tuesday; the HF and NC groups were injected intraperitoneally with the same volume of saline. Before the intervention, the mean FBG of mice in the NC group was 7.08 mmol/L and that of obese mice in the four groups was 7.89 mmol/L. The FBG was within the normal range, indicating that the mice were all nondiabetic simple obese mice. The body weight and food intake of the mice were measured regularly every day. At the end of the animal experiment (Day 7 after the twelfth intervention), all mice were fasted overnight for 12 h, blood was collected from the eyes, and the supernatant was collected after centrifugation at 5000 r/min for 15 min.

#### Indicators and measurement

(1) General condition: daily observation of the mice’s activities, including observation of their appearance, urine, stool, activity, gait, spirit, appetite, and any abnormal conditions.

(2) Determination of body weight, food intake and blood glucose: The body weight of mice was measured regularly every day, the appropriate amount of food was placed in the enclosures and weighed before putting in, and the weight of the remaining feed was weighed the next day (the large pieces of residue in the cage box of each group were weighed and recorded, and the residue crumbs were ignored), and the daily food intake was calculated. Mice were fasted for 12 h each week without the restriction of water intake, and their fasting blood glucose was measured regularly by tail-tip cutting and hand-held glucometer using the next morning for 12 weeks.

(3) Serum biochemical analysis: At the end of 12 weeks intervention, all mice were fasted overnight for 12 H, and they were executed by cervical dislocation after eye blood collection, and the supernatant was preserved after centrifugation at 5000r/min for 15 min. The levels of FBG, INS and CRP were detected, and the level of Homa-IR was calculated according to the steady-state model method formula: Homa-IR index = FBG (mmol/L) x INS (mIU/L) /22.5. The serum chemerin and omentin levels were determined in mice according to the kit instructions.

### Statistical analysis

Analysis was performed using SPSS 22.0. The measurement data were presented as the means ± standard deviation (x ± s); statistical comparisons among groups were performed using one-way analysis of variance (ANOVA) and t-tests; Pearson correlation analysis was used for correlation; *P* < 0.05 was considered statistically significant.

## Results

### General conditions

All three groups of mice in the intervention group had a significant decrease in activity and water intake after being injected with PEX-168 during the first two weeks, especially in the three days after the injection. The inhibitory effect gradually improved with the extension of the intervention time, and the LD group recovered the fastest. During the intervention period, all mice had dark and shiny coats.

### Effect on the intake of mice

**Fig. 1:**
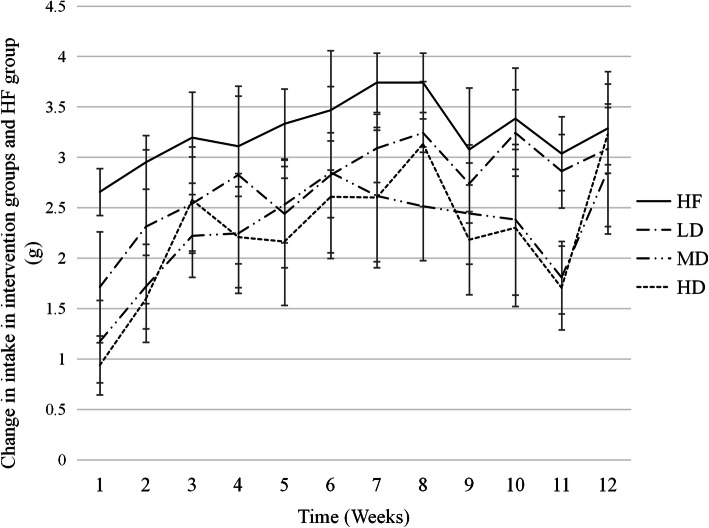
The food intake of each group was compared for 12 weeks. Data are means ± s.d. Values were expressed as a box and whisker with minimum and maximal value. The figures showed the average daily food intake per week in each group

Food intake in the three intervention groups had a significant decrease during the first three weeks. The inhibition of food intake was strongest on the first day of each intervention week, and gradually improved in the second six days. In a word, the food intake suppression effect gradually weakened with the duration of the intervention (Fig. 1). Compared with the HF group, the food intake of the three intervention groups was significantly reduced in the intervention period, and the difference was statistically significant (LD *P *<0.05, MD *P *= 0.004 < 0.01, HD *P *= 0.002 < 0.01). In the late intervention period, the difference in food intake between the HF and LD groups was not statistically significant (*P* > 0.05).

### Effects on body weight, blood glucose, and insulin in mice

The mean weight of the mice in the control group before the start of the experiment was 21.89 g. There was no significant difference in weight between all the obese mice groups (*P* > 0.05). During the intervention period, the body weight of mice in the HF group was relatively stable. After the intervention, the body weight of the mice in the three groups was still higher than that in the NC group, and the difference was statistically significant (*P *< 0.001). Compared with the HF group, the weight of the mice in the three groups had a downward trend, among which the body weight of mice in MD and HD groups decreased significantly and the difference was statistically significant (MD *P *= 0.009 < 0.01, HD *P *= 0.025 < 0.05). The weight loss of mice in the LD group was statistically significant in the first eight weeks (*P *< 0.05) (Tables 1 and 2).

**Table 1:** The average weight at different weeks in each group

Groups	NC	HF	LD	MD	HD
Week 0	21.88±0.46 g	31.33±1.53 g	30.29±1.48 g	31.97±2.88 g	29.90±2.23 g
Week 4	22.38±0.68 g	29.64±1.38 g	28.02±1.47 g	27.00±1.13 g	27.16±1.33 g
Week 8	22.39±0.85 g	31.07±2.40 g	28.42±1.18 g	27.86±1.26 g	28.56±1.24 g
Week 12	23.52±0.81 g	31.57±2.08 g	31.95±1.97 g	28.67±0.83 g	29.04±1.67 g

Data are means ± s.d

**Table 2:** Comparison of weight、FBG、FINS、Homa-IR in each group

Groups	Quantity	Weight (g)	FBG (mmol/L)	FINS (mIU/L)	Homa-IR
NC	*n*=5	23.52±0.90^a^	7.40±0.12^a^	7.70±0.17^a^	2.59±0.05^a^
HF	*n*=6	31.57±2.28	8.33±0.15	10.01±0.10	3.79±0.08
LD	*n*=5	31.95±2.20^b^	8.02±0.15^ab^	8.50±0.28^ab^	3.1±0.14^ab^
MD	*n*=6	28.67±0.91^ab^	8.08±0.17^ab^	8.62±0.24^ab^	3.17±0.13^ab^
HD	*n*=5	29.04±1.87^ab^	7.94±0.21^ab^	8.65±0.25^ab^	3.12±0.07^ab^

^a^*P* < 0.05 vs. the HF group; ^b^*P* < 0.05 vs. the NC group. Data are means ± s.d

After the intervention, the levels of FBG, INS, and Homa-IR of mice in all four groups were significantly higher than those in the NC group, with statistically significant differences (*P* < 0.001). When compared with the HF group, the levels of FBG, INS and Homa-IR of obese mice in the three intervention groups were significantly lower, with statistically significant differences (FBG *P* < 0.05, INS Homa-IR *P *< 0.001). There were no significant differences in the improvement of FBG, INS and Homa-IR in mice between the three intervention groups. In the 10th week of the intervention, a transient hypoglycaemic event occurred in the MD and HD groups, with four and three mice having FBG below 5 mmol/L, with incidences of 67% and 50%, respectively, followed by a return to normal levels

### Effects on CRP, and the adipokines chemerin and omentin in mice

As shown in Table 3, compared with the NC group, there were significant differences in the levels of CRP, chemerin and omentin in the four groups (chemerin and omentin *P* < 0.001, CRP *P* < 0.05). CRP was significantly lower in the MD and HD groups than that in the HF group, and the differences were statistically significant (*P* < 0.05). There was no difference in the improvement between the MD and HD groups (*P* > 0.05). The serum chemerin levels of mice in all three intervention groups were lower than those in the HF group, and the difference was statistically significant (*P* < 0.01). The serum omentin levels of mice in all three intervention groups were higher than those in the HF group, and the difference was statistically significant (*P* < 0.001). There was no significant difference in the improvement of serum chemerin and omentin levels between the 3 groups of mice receiving different doses of PEX-168 (*P* > 0.05).

Insulin resistance in mice was negatively correlated with serum omentin levels (r = -0.836, *P *< 0.001) and positively correlated with serum chemerin levels (r = 0.828, *P *< 0.001).

**Table 3:** Comparison of CRP、Chemerin and Omentin levels in each group

Groups	CRP (ug/L)	Chemerin (ng/L)	Omentin (ng/L)
NC	1327.39±27.05^a^	50.85±1.19^a^	147.76±1.62^a^
HF	1410.48±25.45^b^	56.79±0.85^b^	131.14±3.14^b^
LD	1403.08±35.26^b^	54.65±1.47^ab^(*p*=0.007)	139.67±3.88^ab^(*p*=0.000)
MD	1370.17±31.32^ab^(*p*=0.033)	54.30±1.33^ab^(*p*=0.001)	139.10±3.22^ab^(*p*=0.000)
HD	1370.44±33.90^ab^(*p*=0.042)	54.66±1.02^ab^(*p*=0.007)	137.58±2.83^ab^(*p*=0.000)

^a^*P* < 0.05 vs. the HF group; ^b^*P* < 0.05 vs. the NC group. Data are means ± s.d

## Discussion

### Body weight and food intake

In the present study, low doses of PEX-168 inhibited the progression of obesity in the short term, and medium to high concentrations of PEX-168 inhibited the development of obesity throughout the intervention. The corresponding changes in food intake and body weight also demonstrated that the weight-reducing effect of PEX-168, like other GLP-1 analogues, was mainly through inhibition of intake, which is also consistent with animal experiments with liraglutide. However, the decrease in food intake was unstable during the intervention and was gradually normalized by the mice as the intervention progressed and the inhibition of food intake diminished. This may be because there are other factors that inhibit food intake besides just the feeling of fullness, such as gastrointestinal discomfort. The most common adverse reaction of GLP-1 RA is gastrointestinal reactions, which gradually decrease with a longer dosing time and are significantly dose-dependent[22]. GLP-1 also has a profound inhibitory effect on gastric emptying. The delayed gastric emptying induced by GLP-1 is influenced by a rapid response at the level of vagal activation, and the delay in gastric emptying by GLP-1 is significantly attenuated after long-term administration of GLP-1 analogues[23], which was also consistent with the changes in intake of the intervention groups in this experiment. In addition, PEX-168 is a long-acting preparation with a more sustained hypoglycaemic effect and less effect on gastric emptying, leading to a gradual weakening of the inhibitory effect on food intake.

### Insulin resistance

In this study, the hypoglycaemic effect of PEX-168 on simple obese mice manifested at low doses, and all three doses of PEX-168 inhibited the development of prediabetes and improved insulin resistance in nondiabetic simple obese mice. In Khound’s animal study[24], it was shown that elevated GLP-1 prevented the overproduction of VLDL and improved insulin resistance induced by a high-fat diet in mice, which is consistent with the results of the present study.

However, clinical trials have confirmed the safety of PEX-168 in regulating blood glucose in T2DM patients, and the risk of hypoglycaemia is very low with monotherapy. In this study, PEX-168 was used in nondiabetic simple obese mice, and hypoglycaemic events occurred in the middle- and high-dose groups, indicating that there is a certain risk of hypoglycaemia when PEX-168 intervenes in simple obese mice with normal blood glucose, and the low dose should be considered the starting dose.

### Inflammatory reaction

C-reactive protein, synthesized by the liver, is a sensitive marker of systemic inflammation. It is a nonspecific acute phase reactant that has traditionally been used to detect acute injury, infection and inflammation[25]. Recent studies have shown that diabetes, obesity and elevated levels of CRP, TNF-alpha and leptin are closely associated[26].

In the present study, the level of CRP was significantly elevated in obese mice compared to NC mice, which is consistent with previous reports. The association between obesity and elevated serum CRP levels has been well explained by pathophysiological mechanisms. In this experiment, only the MD and HD groups showed significant improvement in inflammation, and both body weight and the level of CRP in the LD group were not significantly different from those in the HF group, indicating that PEX-168 at medium doses and above significantly reduces body weight in simple obese mice, thereby improving the inflammatory reaction and reducing cardiovascular risk.

### Adipose factor

In this study, the level of serum chemerin was significantly higher and the level of serum omentin was significantly lower in obese mice than in the NC group. The concentration of chemerin was associated with BMI, adipocyte volume and number, and in adult obese patients, body weight was significantly and positively correlated with circulating chemerin levels[27]. Batista assessed the concentration of omentin in obese patients[28], and normal weight subjects showed higher levels of omentin than overweight and obese patients, which is also consistent with the findings of the study.

Interestingly, although the LD group did not lose weight at the end of the intervention, the levels of FBG, INS, Homa-IR, chemerin and omentin were improved, and the improvements were equal across doses. In Yang’s study[29], it was suggested that the GLP-1 analogue liraglutide could improve insulin resistance in high-fat diet-induced obese mice by improving endoplasmic reticulum stress, thereby reducing chemerin levels. K. Tan and Yan pointed out that[16, 30]insulin and glucose could significantly and dose-dependently reduce omentin-1 mRNA and protein levels, and the plasma level of omentin-1 was independently and negatively correlated with fasting insulin and HOMA-IR. K. Tan also pointed out that BMI or WHR is unlikely to be responsible for the decrease in omentin-1 mRNA expression and protein levels in female PCOS patients. In this study, insulin resistance was highly and significantly correlated with both chemerin and omentin, so it can be inferred that the regulation of chemerin and omentin expression by PEX-168 may be related to the hypoglycaemic effect of PEX-168, and the weight-reducing effect of PEX-168 is unlikely to be the reason for the changes in both. This conclusion needs to be demonstrated by further experimental studies.

## Study strengths and limitations

In this study, the weight reduction effect of PEX-168 was first studied and the effects of it on adipokines chemerin and omentin were further explored. PEX-168 has good potential in treating obesity and preventing the development of diabetes. PEX-168 significantly improves the adipokines through its hypoglycaemic effects. These results provide further mechanistic insight into the action of PEX-168 in the treatment of obesity and diabetes.

However, it has been suggested that[11]non-type 2 diabetic patients lose more weight than type 2 diabetic patients when treated with GLP-1 agonists for weight loss, and this could not be confirmed in this experimental design without the inclusion of a diabetic obesity model group.

Due to our initial experimental design, the sample comprised male mice. the results in this animal study pertain only to the male sex. Since it is a novel hypoglycaemic and anti-obesity drug, we did not set a drug control group in this experiment, but to explore the appropriate concentration to achieve positive effects. However, the addition of a drug control group (like liraglutide) would have made the results more visual and clinically meaningful. We call for the inclusion of the drug control group and genders in the further experiment.

The intervention period was not long enough. In clinical trials, it was noted that PEX-168 blood levels reached stability after four weeks of intervention, but in this study the measurements of the mice only started to stabilize at a later stage, so the intervention period should be extended.

## Conclusions

In summary, the data suggest that in nondiabetic simple obese mice, the antidiabetic drug PEX-168 can effectively reduce body weight, improve insulin resistance, reduce the inflammatory reaction, reduce chemerin and increase omentin levels, and prevent the development of prediabetes. PEX-168 regulates the expression of chemerin and omentin probably through its hypoglycaemic effects, and the weight-reducing effect of PEX-168 is unlikely to be the reason for the changes in both. This study may contribute to the guidance of clinically relevant drugs, broaden the treatment of obesity and contribute to the further exploration of the exact mechanism by which PEX-168 regulates adipokines chemerin and omentin. We will improve the experimental design and conduct further prospective clinical studies in the future.

## Data Availability

Data and material would be supplied based on reasonable request. If someone wants to request the data, please email 13,665,278,170@163.com.

## Abbreviations

PEX-168: Polyethylene glycol loxenatide
GLP-1: Glucagon-like peptide-1
GLP-1 RA: Glucagon-like peptide-1 receptor agonist
WHR: Waist-to-hip ratio
Homa-IR: Homeostasis model assessment of insulin resistance
FBG: Fasting blood glucose
INS: Insulin
CRP: C-reactive protein
BMI: Body mass index
PCOS: Polycystic ovarian syndrome
